# Evaluation of the added value of viral genomic information for predicting severity of influenza infection

**DOI:** 10.1186/s12879-021-06510-z

**Published:** 2021-08-10

**Authors:** Nina Van Goethem, Annie Robert, Nathalie Bossuyt, Laura A. E. Van Poelvoorde, Sophie Quoilin, Sigrid C. J. De Keersmaecker, Brecht Devleesschauwer, Isabelle Thomas, Kevin Vanneste, Nancy H. C. Roosens, Herman Van Oyen

**Affiliations:** 1 Scientific Directorate of Epidemiology and Public Health, Sciensano, J. Wytsmanstraat 14, 1050 Brussels, Belgium; 2 Department of Epidemiology and Biostatistics, Institut de Recherche Expérimentale et Clinique, Faculty of Public Health, Université Catholique de Louvain, Clos Chapelle-aux-champs 30, 1200 Woluwe-Saint-Lambert, Belgium; 3 Transversal Activities in Applied Genomics, Sciensano, J. Wytsmanstraat 14, 1050 Brussels, Belgium; 4 Department of Veterinary Public Health and Food Safety, Ghent University, Salisburylaan 133, 9820 Merelbeke, Belgium; 5 National Reference Center Influenza, Sciensano, J. Wytsmanstraat 14, 1050 Brussels, Belgium; 6 Department of Public Health and Primary Care, Ghent University, De Pintelaan 185, 9000 Ghent, Belgium

**Keywords:** Seasonal influenza, Pathogen genomics, High-dimensional data analysis, Predictive modeling

## Abstract

**Background:**

The severity of an influenza infection is influenced by both host and viral characteristics. This study aims to assess the relevance of viral genomic data for the prediction of severe influenza A(H3N2) infections among patients hospitalized for severe acute respiratory infection (SARI), in view of risk assessment and patient management.

**Methods:**

160 A(H3N2) influenza positive samples from the 2016–2017 season originating from the Belgian SARI surveillance were selected for whole genome sequencing. Predictor variables for severity were selected using a penalized elastic net logistic regression model from a combined host and genomic dataset, including patient information and nucleotide mutations identified in the viral genome. The goodness-of-fit of the model combining host and genomic data was compared using a likelihood-ratio test with the model including host data only. Internal validation of model discrimination was conducted by calculating the optimism-adjusted area under the Receiver Operating Characteristic curve (AUC) for both models.

**Results:**

The model including viral mutations in addition to the host characteristics had an improved fit (${X}^{2}$=12.03, df = 3, p = 0.007). The optimism-adjusted AUC increased from 0.671 to 0.732.

**Conclusions:**

Adding genomic data (selected season-specific mutations in the viral genome) to the model containing host characteristics improved the prediction of severe influenza infection among hospitalized SARI patients, thereby offering the potential for translation into a prospective strategy to perform early season risk assessment or to guide individual patient management.

**Supplementary Information:**

The online version contains supplementary material available at 10.1186/s12879-021-06510-z.

## Introduction

Influenza is ranked as the infectious disease with the highest impact on population health in the Burden of Communicable Diseases in Europe in the period 2009–2013 [1]. Influenza types A and B are the predominant types causing disease in humans [2], of which type A viruses exhibit the greatest genetic diversity, infect the widest range of host species and cause the vast majority of severe disease in humans [3]. Influenza A viruses mainly circulating worldwide in humans are subtypes A(H1N1) and A(H3N2) [2, 4].

Severity is one of the critical parameters for influenza monitoring. Following the A(H1N1) pandemic in 2009, the World Health Organization (WHO) and the European Center for Disease Prevention and Control (ECDC) recommended to implement a hospital-based surveillance of Severe Acute Respiratory Infections (SARI) to monitor the severity of influenza infection and the virulence of circulating strains, upon which several countries have strengthened their surveillance of severe influenza infections in order to rapidly detect new variants and assess their population impact [5–10]. In Belgium, a SARI surveillance network has been implemented since 2010 and includes six sentinel hospitals spread over Belgium [11]. The main goal of this surveillance is to contribute to the early detection of signals of seasonal influenza severity. Clinical monitoring of severity is based on well-defined severity indicators (i.e. complications), including treatment in an intensive care unit (ICU), acute respiratory distress syndrome (ARDS), extracorporeal membrane oxygenation (ECMO), invasive respiratory support, and death.

The severity of seasonal influenza depends on the virus, host factors, and other factors such as access to care [12]. Host characteristics such as age and several comorbidities [13–15], including diabetes, chronic lung condition, cardiovascular disease, hepatic disease, hematologic condition, obesity, chronic renal failure, neurological condition, and suppressed immune function [12] have been identified as risk factors for severe seasonal influenza [13]. Disease severity can however also be related to characteristics of the virus itself. For example, subtype A(H3N2) caused more deaths than A(H1N1) [16, 17] and influenza B infections [17, 18], and is especially severe in the elderly [3, 19]. Influenza evolves continuously via reassortments and point mutations that can influence host specificity and viral pathogenicity. The influenza genome is subject to high mutation rates (antigenic drift) due to the lack of proofreading of the influenza viral RNA-polymerase [20]. Newly evolved mutations can help the virus to evade the host immune system, and therefore be positively selected and passed on to the next generation [21]. Hemagglutinin (HA) and neuraminidase (NA), located on the surface of the virion, are the most studied proteins in influenza virulence and antiviral resistance, as these proteins are involved in the host immune response and are more likely to mutate [22–25].

Until recently, public health laboratories mainly relied on Sanger sequencing of the HA1 region of the HA gene to characterize influenza viruses, which only partially covers one of the eight RNA segments of the viral genome [26]. Next-generation sequencing (NGS) allows whole genome sequencing (WGS) of all eight segments of the influenza genome in one single reaction through a massively parallel sequencing set-up [27]. Consequently, WGS offers greater resolution for genetic characterization compared to Sanger sequencing of the HA segment. Previously, we demonstrated that considering the whole genome rather than solely the HA segment substantially improved phylogenetic classification [28]. Additionally, WGS enables the identification of reassortment events [28, 29], the analysis of minor genetic variants in the viral RNA quasispecies population, and the detection of mutations in all segments of the genome potentially related to drug resistance, virulence or other patient characteristics [30–34]. Consequently, crucial information can be missed when considering solely a sub-region of the genome obtained through the more traditional Sanger sequencing of the HA1 region. In order to make genomic data truly useful, it should however be combined with epidemiological data, and data sharing should be facilitated through global surveillance initiatives [35]. For instance, the Global Initiative on Sharing All Influenza Data (GISAID) promotes the international sharing of all influenza virus sequences, and related clinical and epidemiological data [36].

High-throughput technologies such as NGS generate high-dimensional data with many predictors, as every genomic position constitutes a variable, compared to the typically limited number of independent observations (i.e., patients or samples). An integrated data set, which combines the ‘classical’ host characteristics (including age, gender, vaccination status, and co-morbidities) with genomic viral characteristics (i.e., mutations), can enable building a predictive model for the severity of influenza infection among SARI patients. Modeling severe outcomes of influenza infections has however mainly focused on host characteristics as potential risk factors using conventional multivariable regression methods [17, 37–42]. Although many mutations in the influenza genome have been linked to virulence [43–46], studies in a clinical or public health context that aim to incorporate genomic information remain scarce [19, 32]. This is partly due to numerous issues associated with employing high-dimensional data for predictive modelling, such as false discoveries and vulnerability to overfitting, also referred to as the ‘curse of high-dimensionality’, resulting in standard regression methods to perform poorly on high-dimensional datasets [47, 48]. Regularization methods provide an alternative strategy that aim to mitigate this by creating a linear regression model that is penalized for having too many variables in the model by adding a constraint in the equation (also called shrinkage) [49, 50] that shrinks coefficient values towards zero for less contributive variables, thereby allowing variable selection [51]. Such penalized regression methods are commonplace for variable selection in high dimensional studies focusing on human genetic data [52–56], but have to the best of our knowledge not yet been applied to an infectious disease such as influenza.

Here, we evaluate the added value of applying genomic data as additional information to complement the existing surveillance system. Incorporating viral WGS information could potentially result in a better understanding of the different (currently unknown) factors that impact disease severity [19]. Our study therefore aims to find the combination of predictors for the severity of influenza infection among patients hospitalized for SARI, and to assess the relevance of adding genomic viral data for increasing the performance of predictive modelling approaches.

## Methods

### Data collection

The SARI case definition is an acute respiratory illness with fever of ≥ 38 °C, cough or dyspnea, and requiring hospitalization for at least 24 h. Surveillance is carried out within six sentinel hospitals and only during the epidemic period of seasonal influenza. All hospital wards (including pediatric and adult units) collect both clinical data and nasopharyngeal swabs from every patient who corresponds to the SARI case definition. Samples and clinical forms are sent to the Belgian National Reference Center (NRC) of Influenza [11]. Among SARI patients, a severe influenza infection is defined based on the presence of at least one severe complication, i.e. stay in ICU, ARDS, ECMO, invasive respiratory support, or death, as recorded on the clinical forms. Data and accompanying samples from this existing surveillance system, i.e. routinely collected health data, were used for the current study. All methods were performed in accordance with relevant guidelines and regulations. The protocol of the SARI surveillance has been submitted for approval to the Sectorial committee for social security and health (i.e. private life commission). The Sectorial committee authorised the communication of private data in the frame of this surveillance during its deliberation n° 15/043 of the 16 June 2015.

### Study design

A population-based case–control study was performed. Cases and controls were chosen from a larger retrospective ‘case-series’ of the 2016–2017 SARI hospitalized population. The influenza 2016–2017 season was used as this was the most recent season for which data collection was completed at the point of initiating the study. Samples from 1422 SARI patients were sent to the NRC during this season. Samples of 563 patients tested positive for influenza, of which 526 were positive for Influenza A(H3N2). Patients with a co-infection (n = 131), including viral co-infections as identified by the NRC using multiplex real-time quantitative polymerase chain reaction (RT-qPCR) and bacterial co-infections (e.g. bacterial pneumonia) as detected in the hospital, were excluded. The remaining 395 patients formed the potential case–control study population. Samples to be sequenced were selected based on the quantification cycle (Cq) values, which is a semi-quantitative measure of the amount of virus DNA in a clinical specimen (the lower the Cq value, the more product was produced by a PCR). With high Cq values, the viral load might be insufficient for sequencing.

**Fig. 1:**
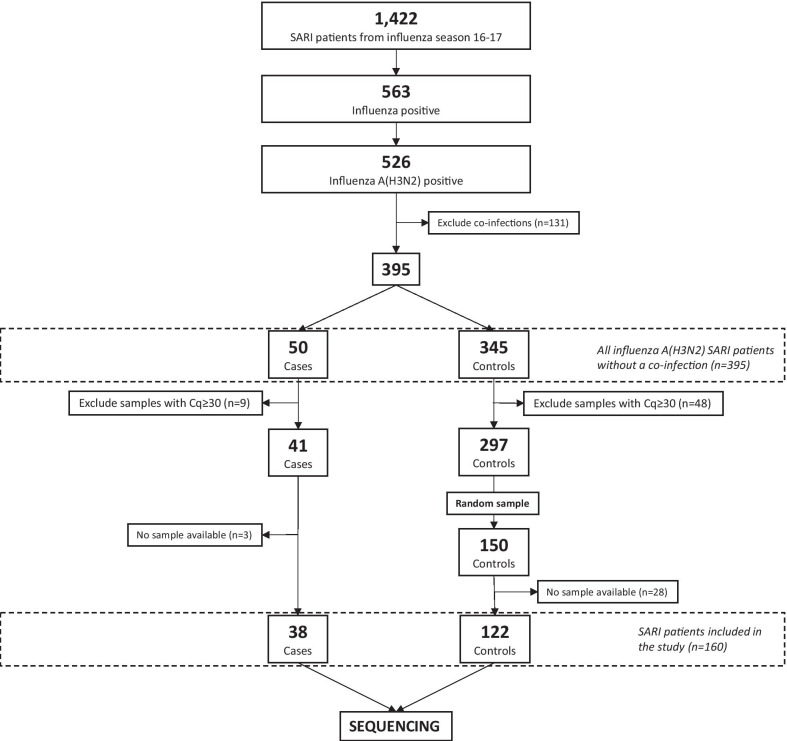
Selection of severe acute respiratory infection (SARI) samples from a larger retrospective ‘case-series’ of the 2016–2017 SARI hospitalized population using a case–control sampling approach. SARI patients were categorized in two groups: **a** cases, defined as SARI patients with a severe influenza infection based on the presence of at least one severe complication, i.e. stay in intensive care unit (ICU), acute respiratory distress syndrome (ARDS), extracorporeal membrane oxygenation (ECMO), invasive respiratory support, and death, and **b** controls, defined as SARI patients without one of these severe complications. Cq (quantification cycle) = relative measure of the concentration of target in the PCR reaction

Cases were considered as SARI patients with a severe influenza infection (i.e. presence of at least one severity indicator) as defined in the previous section. Of all SARI patients with a severe influenza infection (n = 50), 9 had a Cq ≥ 30 and for 3 there was no accompanying sample available, resulting in 38 included cases. For the control study population (i.e. SARI patients without a severe complication, n = 345), 48 were excluded based on a high Cq value (Cq ≥ 30). From the remaining 297 patients, 150 controls were randomly selected to aim for a case–control ratio of at least 1:3. From these, 122 controls had samples available for sequencing. Correspondingly, samples from a total of 160 patients (38 cases and 122 controls) were used for sequencing. An overview of the selection process is presented in Fig. 1.

### Whole genome sequencing and identification of mutations

After RNA extraction from the clinical samples, reverse transcription polymerase chain reaction (RT-PCR) was performed with three universal primers to amplify the eight segments of the influenza A genome. All of these RT-PCR products were purified, the presence of the segments was verified, and the DNA concentration of each sample was measured. NGS was performed on the Illumina MiSeq (chemistry v3, 2 × 250 bp) using the Nextera XT DNA Sample Preparation Kit for library preparation. Full details on RNA isolation, PCR amplification and WGS have been described previously [28]. All generated WGS data have been deposited in the NCBI Sequence Read Archive (SRA) under accession number PRJNA615341. Afterwards, the consensus genome sequence was obtained for every sample as described previously [28] and are available in the GISAID database as isolates ID EPI_ISL_415292 to EPI_ISL_415452. Nucleotide mutations, i.e. Single Nucleotide Polymorphisms (SNPs), were identified by aligning the consensus genome sequence for every sample to a reference genome (strain A/Hong Kong/4801/2014, GISAID accession number EPI_ISL_198222). Untranslated regions were stripped on both sides retaining only the protein-coding parts on which the nucleotide enumeration was based. Mutations with an overall frequency of occurrence over all samples < 5% or > 95% were discarded. A detailed description of these procedures, including the identification of genetic subgroups, is provided by Van Poelvoorde et al. [34].

### Host characteristics

The dataset of host characteristics obtained through the clinical forms included the sampling period (beginning, middle, or end of the 2016–2017 epidemic period), age, gender, vaccination status, and co-morbidities (chronic respiratory condition, chronic cardiovascular condition, renal insufficiency, hepatic insufficiency, immunocompromised condition, pregnancy, asthma, neuromuscular condition, diabetes, and obesity).

### Data exploration and univariate analysis

All analyses were performed using R software (R version 3.6.0) [57]. Host characteristics were described for the 160 included SARI patients, stratified per severity status (cases versus controls). Univariate descriptive comparisons of host characteristics between severe (cases) and non-severe (controls) patients used the Fisher’s exact test for categorical data, and the Wilcoxon rank sum test for continuous data. Likewise, for descriptive purposes a Fisher’s exact test was used to assess univariate associations between the mutations and severity of infection. Multiple testing correction was conducted by applying the Benjamini–Hochberg method [58]. Explorative analyses and visualization of the multivariable data were performed using principal components analysis (PCA), separately for the host characteristics and genomic data. To identify potential selection bias, baseline characteristics were compared between cases and controls within the set of SARI patients included in the study (n = 160), and between cases and controls within the total cohort of influenza-positive A(H3N2) SARI patients without a co-infection within the 2016–2017 season (n = 395).

### Model building

**Fig. 2:**
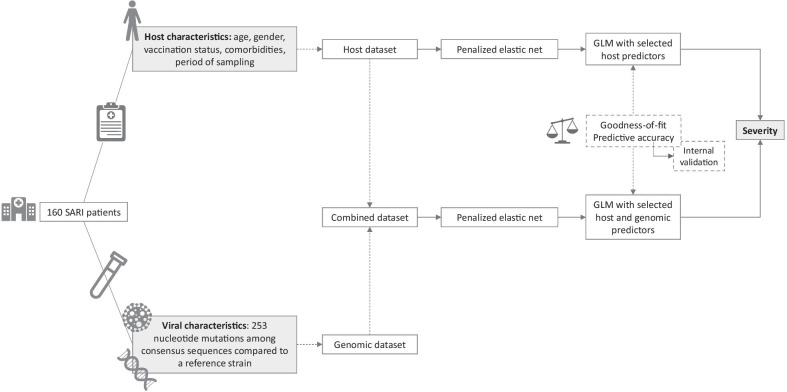
Overview of the predictive model building process to compare a model including variables obtained from a combined dataset (i.e. host characteristics and viral characteristics) and a model including variables from the host characteristics dataset. *SARI*  severe acute respiratory infection, *GLM*  generalized linear model

The outcome was defined as a binary variable: severe influenza infection based on the presence of at least one severity indicator (cases) and non-severe influenza infection based on the absence of severity indicators (controls). Elastic net regression was used as a regularization method to build a predictive model for severity that is able to handle high-dimensional datasets [59]. This penalized model was used to select predictor variables for severity from the combined dataset of mutations and host characteristics described previously. The analysis was conducted using the package ‘glmnet’ version 2.0–18 of the R software that allows to fit penalized regression models with alpha values between 0 and 1 to fit an elastic net model. Lambda refers to the λ penalty parameter. To estimate these model parameters, eleven models were fitted with alpha ranging from 0 to 1 in steps of 0.1. Lambda was chosen using five-fold cross validation for each separate model. The cross-validated mean squared error (CVMSE) of the different models was compared to select the optimal alpha estimate. The models were bootstrapped 300 times and only those variables that were selected by the elastic net regression in at least 80% of the models were retained as predictors. The exact cut-off to retain the predictors was selected based on the Akaike Information Criteria (AIC) [60] of the resulting models. A logistic regression model with those predictors was subsequently constructed to estimate adjusted odds ratios (OR) and the corresponding 95% confidence intervals (CI). Potential (multi)collinearity of the selected predictors was assessed based on the variance inflation factor (VIF). Additionally, interactions within the combined model between the selected mutations and host characteristics were explored one-by-one by comparing models with and without the interaction term by a likelihood-ratio test (LRT). In addition, the potential interaction effect of the genetic subgroups (clades) on the association between the selected mutations and severity was assessed to take into account the viral genetic background, as suggested by Van Poelvoorde et al. [34]. An elastic net regression model was fitted independently on a dataset only including the host characteristics using the same approach as for the combined model. An overview of the model building process is presented in Fig. 2.

### Model comparison

The added value of including genomic data was assessed by comparing the combined model and the host model in terms of the goodness-of-fit and predictive accuracy. Goodness-of-fit of the nested models was assessed with the AIC [60] and the LRT. The predictive accuracy was estimated using the area under the receiver operator characteristic (ROC) curve (AUC) that indicates how well the models discriminate between severe and non-severe influenza infections among SARI patients. In addition, the integrated discrimination improvement (IDI) index was calculated as a measure of the incremental value of the viral genomic predictors [61]. Cut-off points to determine the sensitivity and specificity of the models were selected using the Youden index [62, 63]. Internal validation of both models was assessed by calculating the optimism-adjusted AUC obtained through 200 bootstrap resamples [64], accounting for overfitting of model parameters. The bootstrap resampling process starts with fitting models in a bootstrap sample of the same size as the original sample, selected with replacement from the original sample, and then evaluates model performance in both the bootstrap resample and original sample. The difference between the bootstrap and test performances is referred to as optimism. The optimism (averaged over 200 bootstraps) is then subtracted from the apparent performance (i.e. the performance in the original sample) to obtain the optimism corrected estimated performance [65, 66]. The rationale supporting this internal validation approach can be found in the Additional file 1: Appendix A. The overall model performance and calibration were assessed by calculating the Brier score (distance between fitted and actual values) and by performing the Hosmer–Lemeshow test (goodness-of-fit) [67], respectively.

In addition to the approach described above, another data analysis scenario based on stepwise regression was explored and is presented in the Additional file 1: Appendix B to increase transparency and to assess the impact of methodological choices on the final conclusions [68].

## Results

### Data exploration and univariate analyses

Baseline characteristics (sampling period, age, gender, vaccination status, and co-morbidities) of the 160 included SARI patients stratified per severity status are presented in Table 1. Out of 160 included patients, 38 were cases as they had at least one severe complication and were therefore considered as severe infections (i.e. ‘severe SARI patient’). The median age of all included SARI patients was 77.5 years, and was equal between cases and controls. The sampling period within the influenza season was also comparable between cases and controls. Severity was significantly associated with the presence of a chronic cardiovascular condition (p = 0.047), a chronic respiratory condition (excluding asthma) (p = 0.02), renal insufficiency (p = 0.02), and immunocompromised condition (p = 0.02). The vaccination status was not significantly different between cases and controls, but vaccination status was unknown for 70 out of 160 patients. To assess potential selection bias, baseline characteristics were also compared between all cases (i.e. patients with a severe infection) (n = 50) and the full subset of potential controls (n = 345) among all influenza-positive A(H3N2) SARI patients without a co-infection within the 2016–2017 season (n = 395) (see Additional file 1: Appendix C). No selection bias was detected as severity was associated with the same host characteristics as reported for our study population.

**Table 1:** Characteristics of patients hospitalized for severe acute respiratory infection (SARI) (n = 160), stratified per severity status, i.e. severe influenza A(H3N2) infections (cases) and non-severe influenza A(H3N2) infections (controls), Belgium, Influenza season 2016–2017

	Severe SARI patients (i.e., cases) (n = 38)		Non-severe SARI patients (i.e., controls) (n = 122)		p value^a^
	Median + IQR	Total	Median + IQR	Total	
Age (years)	77.5 (67.5–82)	38	77.5 (61–84)	122	0.94
Sampling period (weeks)	5 (4.25–6)	38	5 (4–6)	122	0.31
	*Proportion*	*Total*	*Proportion*	*Total*	
Males	0.54	37	0.48	119	0.58
Vaccinated	0.53	19	0.34	71	0.18
Chronic cardiovascular condition	0.47	38	0.29	122	0.047
Chronic respiratory condition	0.39	38	0.20	122	0.02
Renal insufficiency	0.34	38	0.16	122	0.02
Hepatic insufficiency	0.03	38	0.04	122	> 0.99
Immunocompromised condition	0.26	38	0.10	122	0.02
Pregnant	0.00	38	0.02	122	/
Asthma	0.00	38	0.06	122	/
Neuromuscular condition	0.11	38	0.12	122	> 0.99
Diabetes	0.18	38	0.15	122	0.61
Obesity	0.08	38	0.11	122	0.76

*SARI* severe acute respiratory infection, *IQR* interquartile range

^a^Wilcoxon rank sum test applied for medians and Fisher’s exact test applied for proportions

A total of 253 nucleotide mutations were identified among the 160 consensus sequences of the sequenced samples. The association between this genome-wide SNP panel (n = 253) and severity was tested using the univariate Fisher’s exact test. A total of 25 mutations had a p-value < 0.05 (see Additional file 1: Appendix D). As we carried out one Fisher’s exact test for every mutation (n = 253), we corrected for multiple testing using the false discovery rate (FDR). The Manhattan plot in Additional file 1: Appendix E shows that after controlling the FDR at 5%, no significant associations could be identified between mutations and severity.

For explorative purposes, visualization of the multivariable data using principal component analysis (PCA) is presented in Additional file 1: Appendix F.

### Model building

Elastic net regression was used to select variables from the combined dataset of host characteristics and viral mutations, and separately from the host dataset. Vaccination status was not included as a variable, as it was unknown for 70 patients. The elastic net regression model selected the following predictors from the dataset containing only host characteristics: chronic respiratory condition, chronic cardiovascular condition, renal insufficiency, immunocompromised condition, and asthma. The parameter estimates of the elastic net model with host characteristics as input were alpha = 0.2 and lambda = 0.063. As the coefficient of the asthma variable resulted in an inflated standard error due to quasi-perfect separation and as it did not significantly improve the goodness-of-fit following a LRT, it was removed from the final host model. A logistic regression model was fitted with the remaining four host characteristics (chronic respiratory condition, chronic cardiovascular condition, renal insufficiency, and immunocompromised condition) resulting in an AIC of 168.63.

When providing both host characteristics (n = 13) and viral mutations (n = 253) in a combined data set as input for the elastic net regression, the parameter estimates were alpha = 0.5 and lambda = 0.011. The same four host characteristics were selected for the combined model as for the host model (chronic respiratory condition, chronic cardiovascular condition, renal insufficiency, and immunocompromised condition). Sampling period was initially selected by the model selection process but removed manually from the combined model in order to arrive at the same set of host characteristics in accordance with the host model. This was justified by the fact that sampling period did not significantly improve the fit of the model based on the LRT, as well as the fact the AIC was lower when not including sampling period in the model. In addition to the four host characteristics, the following three mutations were selected: PA T135C, PA A1475G and NS A323C. No (multi)collinearities between the covariates were detected. No interactions between the viral and host characteristics could be identified. In addition, there was no interaction effect of the genetic subgroups (clades) on the association between the selected mutations and severity. A logistic regression model was fitted containing the four host characteristics (chronic respiratory condition, chronic cardiovascular condition, renal insufficiency, and immunocompromised condition) and the three mutations (PA T135C, PA A1475G, and NS A323C) resulting in an AIC of 162.60. The adjusted ORs and associated CIs of the selected variables within the host and the combined model are presented in Table 2.

**Table 2:** Host and viral characteristics (mutations) selected for predicting severity of influenza A(H3N2) infection among patients hospitalized for severe acute respiratory infection (SARI) (n = 160), Belgium, Influenza season 2016–2017

	Host model (AIC = 168.6)		Combined model (AIC = 162.6)	
Variable^a^	Odds ratio^b^	95% CI^b^	Odds ratio^b^	95% CI^b^
Chronic respiratory condition	2.16	[0.93, 4.95]	2.76	[1.13, 6.76]
Chronic cardiovascular condition	2.05	[0.91, 4.64]	1.96	[0.83, 4.64]
Renal insufficiency	1.94	[0.78, 4.64]	1.75	[0.68, 4.40]
Immunocompromised condition	3.00	[1.09, 8.19]	3.35	[1.16, 9.76]
PA T135C	–	–	0.12	[0.01, 0.69]
PA A1475G	–	–	2.55	[0.49, 13.00]
NS A323C	–	–	3.32	[0.91, 11.91]

^a^The variables were selected by penalized elastic net regression and fitted as predictors in a logistic regression model

^b^The mean adjusted odds ratio’s and accompanying 95% confidence intervals (CI) are based on the results of the logistic regression model

### Model comparison

Likelihood ratio tests were used to compare the goodness-of-fit between the nested models. The combined model including the 3 mutations and 4 host characteristics had a significantly improved fit compared to the host model only including the 4 host characteristics (${X}^{2}$ = 12.03, df = 3, p = 0.007). The host model resulted in a Brier score (distance between fitted values and actual values) of 0.161, whereas the combined model resulted in a Brier score of 0.145. A lower Brier score indicates more accurate predictions. The Hosmer–Lemeshow goodness-of-fit test resulted in a p-value of 0.959 for the host model, and a p-value of 0.781 for the combined model, indicating no evidence of poor fit.

The predictive accuracy was assessed by the AUC that indicates how well the models discriminate between severe and non-severe SARI influenza infections independently from the exact decision threshold employed. The apparent AUC (see Additional file 1: Appendix G) increased from 0.700 [0.602, 0.799] to 0.773 [0.684, 0.861] when adding the 3 mutations to the model containing host characteristics (p = 0.015). The discrimination slope increased, yielding an IDI of 0.077 [0.039, 0.116] (p < 0.001). After choosing an appropriate objective classification threshold based on the Youden index (0.26 for the host model and 0.25 for the combined model), the host model resulted in an apparent sensitivity of 53% and specificity of 80%, whereas this was respectively 74% and 72% for the combined model. Boxplots of the fitted values are presented in Additional file 1: Appendix H. Internal validation of the predicted accuracy was assessed by optimism-adjustment, which was obtained through 200 bootstrapped resamples (see Table 3). The optimism-adjusted AUC increased from 0.670 to 0.732 when adding viral genomic information (i.e. the three mutations) to the model. The combined model had an optimism-adjusted sensitivity and specificity of 68% and 70% respectively, whereas this was 48% and 79% for the host model.

**Table 3:** Apparent and bootstrap (using 200 resamples) optimism-adjusted measures of accuracy for the combined and host model predicting severity of influenza A(H3N2) infection among patients hospitalized for severe acute respiratory infection (SARI) (n = 160), Belgium, Influenza season 2016–2017

	Host model			Combined model		
	Apparent	Optimism	Corrected	Apparent	Optimism	Corrected
AUC	0.700	0.030	0.670	0.773	0.041	0.732
Sensitivity	0.526	0.044	0.482	0.737	0.058	0.679
Specificity	0.803	0.015	0.788	0.721	0.018	0.703

Approximately similar conclusions could be drawn when adopting an alternative approach consisting of a prioritization step of variables following univariate analysis and subsequent stepwise regression (see Additional file 1: Appendix B).

A genomic model built from a dataset only including mutations was also evaluated and resulted in a lower performance based on AUC than both the combined and host models, suggesting that the increased performance of the combined model was due to incorporating data from both the clinical forms and genomics rather than genomics data on itself (see Additional file 1: Appendix I).

## Discussion

As recommended by WHO and ECDC, severity of influenza infection and the virulence of circulating strains is monitored through the implementation of a hospital-based surveillance of SARI. This study assessed the added value of viral sequence data obtained through WGS to complement “traditional” clinical data for predicting a severe influenza infection among hospitalized SARI patients. A penalized elastic net logistic regression was used to fit models to predict influenza infection severity using either only host characteristics obtained from the clinical forms (the ‘host model’), or alternatively an integrated dataset containing both host characteristics and viral genomic data (i.e. 253 nucleotide mutations) as potential predictors (the ‘combined model’). The elastic net method effectively sets the less contributive model variables to zero thereby allowing variable selection. For both models, such variable selection was employed to find the combination of predictors that optimized the overall model fit. Following subsequent model comparison, the goodness-of-fit increased significantly when adding the selected viral mutations (PA T135C, PA A1475G, and NS A323C) to selected host characteristics (chronic respiratory condition, chronic cardiovascular condition, renal insufficiency, and immunocompromised condition). This indicates that adding viral mutations presents a significant improvement over the more parsimonious host model. Moreover, the optimism-adjusted predictive accuracy was higher for the combined model (AUC = 0.732) compared with the host model (AUC = 0.670). The higher the AUC, the better the model is at distinguishing between severe and non-severe influenza-infected hospitalized patients. Following objective classification threshold selection using the Youden index, the optimism-adjusted sensitivity increased by 20% at only a cost of 9% in specificity.

Predictors for severity selected from the host characteristics through the elastic net approach for both the host and combined models include chronic respiratory condition, renal insufficiency, immunocompromised condition, and chronic cardiovascular condition, in agreement with previous studies [12, 13, 17, 69]. Age was also indicated as an important risk factor for influenza severity following a large systematic review [13], but was not a strong predictor for influenza severity within our population of hospitalized patients. However, these other studies often define severity as requiring hospital admission, while in the current study we evaluated severity among hospitalized patients based on clearly defined severity indicators. Three nucleotide mutations were selected on top of these four host characteristics in the combined model: PA T135C, PA A1475G, NS A323C. Since these mutations are located on the PA (polymerase acid protein) and NS (non-structural protein) segments, Sanger sequencing of only the HA segment as traditionally performed for influenza surveillance would not have identified them, demonstrating the added value of characterizing the other genome segments as well because they can also be good predictors for severity [19, 28, 34, 43]. It should be noted that the choice of reference genome used to identify mutations can potentially have implications. A genomic position with predictive ability present in the reference will not be picked up as only positions that are different from the reference are propagated. Consequently, the more distant the reference strain the more mutations that will be detected.

It has been suggested that mutations in the haemagglutinin (HA), non-structural protein 1 (NS1) and polymerase basic protein 2 (PB2) of influenza viruses might be associated with disease severity [19, 43–46]. The mutations PA T135C, PA A1475G, and NS A323C included in the combined predictive model have, to the best of our knowledge, not been described before in relation to influenza pathogenicity. The NS A323C mutation provokes an amino acid change (Lysine into Threonine) in the NS1 protein. The NS1 protein has been implicated in pathogenicity by playing a role in the evasion of the innate immune response [70–72]. The PA A1475G mutation results in an amino acid change in the PA protein (Lysine into Arginine). The function of the PA subunit is less well defined [73, 74] but plays an essential role in viral RNA transcription and replication. PA T135C is a synonymous mutation, i.e. did not result in an amino acid change in the encoded protein, but it has been demonstrated previously that natural selection can also act on synonymous sites [75, 76]. Nucleotide mutations may also influence pathogenicity of influenza viruses [77], as they can affect packaging, transcription and translation of the virus, interfere with the hosts’ immune response [78–82], and can be co-selected with other sites, supporting our approach of incorporating mutations at the nucleotide rather than amino acid level as potential predictors for severity. The PA T135C mutation would not have been detected as a predictor of severity when only looking at the amino acid level. Furthermore, it should be stressed that selection of predictive mutations does not necessarily imply a causal association with severity. A causal analysis would require a thoughtful selection of confounders. However, the objective of the current study was not to identify individual risk factors that are causally related to an outcome by a direct or indirect effect, but rather to find a combination of factors that best predicts the severity of a current or future diagnosis [83]. When the interest lies exclusively on outcome prediction, one may want to select any variable that, when included as covariate in the model, improves its predictive ability [84]. This explains why the predictive modelling could detect mutations that were not picked up by univariate analysis, after correction for multiple testing.

Strengths of the currently existing SARI surveillance system in Belgium are the well-defined severity indicators, and the absence of sampling bias as a swab is taken for every patient corresponding to the case definition. Furthermore, no selection bias was detected when comparing baseline characteristics between our study population and the SARI database population. Also, as six sentinel hospitals are participating in the surveillance, the data are considered to be more generalizable compared to single-center studies. Limitations include that the quality and completeness of the data cannot always be guaranteed when retrospectively analyzing clinical data from a routine surveillance system [85]. For example, the absence of pre-existing immunity is considered as an important risk factor for influenza disease severity [86]. Consequently, adding information on host immunological status, such as vaccination history, to the model might significantly improve the predictions. The added value of genomic information was assessed by comparing two hierarchical models: a model with host data only and the same model with viral genomic data. If pre-existing immunity plays a role in the severity of influenza, it is expected to induce a bias in both models but not on the difference. Nevertheless, the current prediction model was built using real-life patient data collected as part of a routine surveillance system where vaccination status was missing for 44% of the included patients and therefore not considered as a predictor in the model. When this predictive model would be applied in a real-life setting, it would face the same limitations. Another limitation is the relatively small sample size. Several cross-validation and bootstrapping steps were added to the analysis to avoid an overfitting problem.

A case–control sampling approach was used to select an appropriate subsample of individuals from a larger retrospective ‘case-series’ study population (i.e. routinely collected surveillance data) to ensure that WGS was only performed for as many samples as necessary to avoid costly sequencing in large cohorts, and is more efficient than sampling a random population subset that could by chance include some cases. It is therefore more efficient to utilize all available cases (especially when the outcome is relatively infrequent), and randomly select a number of controls for every case from the remaining population. There exists an upper limit on statistical power if only a limited number of cases are available so that collecting more controls to increase the sample size will not add statistical power once past a certain level [87]. The power gained for case:control ratios above 1:3 or 1:4 is likely poor compared to the additionally required workload [88]. Additionally, controls were not matched to cases, as we aimed to analyze all variables as potential risk factors [89].

Viral genomics has to date only seen limited direct use in clinical or public health practice for predicting infection severity [19, 44], which can partly be explained by its larger cost and difficulty of collecting samples compared to collecting the traditionally employed clinical data. Information on the pathogen genome however increases understanding of disease severity. Broberg et al. [90] highlighted the importance of reporting influenza sequence data along with associated clinical and epidemiological information to improve understanding of factors that may increase the risk of severe influenza. Furthermore, ECDC recommends notification of influenza cases in combination with genetic analysis, and has prioritized influenza for further integration of molecular typing and full genome sequencing into European level surveillance activities and epidemic preparedness [91]. According to a survey conducted by ECDC in 2017, 8 EU/EEA countries use WGS for first or second-line typing for routine surveillance and outbreak investigations of human influenza virus, while 20 countries indicated that they do not use WGS for any public health operations related to influenza. However, 14 countries indicated that the implementation of WGS for human influenza virus is planned by 2019 [92]].

We demonstrated that adding viral genomic information to a predictive model on top of standard host characteristics, provides a more complete view on the predictors of severity of influenza infection, and subsequently increases model performance. A potential limitation of the current approach for implementation into routine practice, is that the modelling approach cannot be fully automated and requires expert decisions and interventions at several steps. Furthermore, given the substantial genetic variation in the influenza genome and its quick mutation rate, the viral model parameters (i.e. selected mutations) should be considered as dynamic rather than fixed. Still, results could be accumulated over multiple influenza seasons to construct a database of mutations with predictive ability. More investigation is needed to understand how such an approach can be translated into public health practice. From a research perspective, the identified mutations are ideal candidates for additional investigations by molecular biology-based approaches to examine if they potentially affect severity. Although being careful against insinuating a causal relationship, this approach would reduce the overall size of genomic positions to be investigated. The added value of other information from the viral genome, such as the detection of reassortment events and the analysis of minor genetic variants in the viral RNA quasispecies population, for predictive modeling of influenza severity should also be further investigated in the future.

At the public health level, a better understanding of the different factors predicting severity, including viral mutations, could serve public health authorities by estimating influenza severity at the beginning of the season (i.e. early season risk assessment [26]). The collection of a sufficient number of samples at the beginning of the season allowing to perform our proposed predictive modelling strategy, would likely require international collaboration between multiple countries, in line with the objectives of GISAID [36]. Information concerning influenza severity could assist public health authorities on advising precautionary measures and/or hospital recommendations. Furthermore, the predictive model could potentially be useful for individual patient care. As SARI cases are sampled at hospital admission, information from the model, if provided in real-time, could potentially allow the identification of people at risk of progressing to severe disease by allowing better patient management and treatment (e.g. administration of antivirals).

## Conclusion

This retrospective study demonstrated the added value of incorporating viral genomic information on top of traditional clinical data for the prediction of severe influenza A(H3N2) infections among hospitalized patients. This approach may allow potential translation into a prospective strategy for surveillance purposes and patient management of influenza infections, and eventually other respiratory viruses.

## Supplementary Information

**Additional file 1. Appendix A**: Internal validation. **Appendix B**: Exploration of other data analysis scenarios. **Appendix C**: Comparison baseline characteristics to assess selection bias. **Appendix D**: List of 25 mutations with an unadjusted p-value below 0.05. **Appendix E**: Manhattan plot. **Appendix F**: Principle Component Analysis (PCA). **Appendix G**: Apparent Area Under the ROC curve. **Appendix H**: Boxplots of the fitted values for the host and the combined model. **Appendix I**: Genomic model.

## Data Availability

All generated WGS data have been deposited in the NCBI Sequence Read Archive (SRA) under accession number PRJNA615341 (https://www.ncbi.nlm.nih.gov/bioproject/PRJNA615341). The consensus genome sequences are available in the GISAID database as isolates ID EPI_ISL_415292 to EPI_ISL_415452. Further requests regarding the clinical dataset analyzed during the current study might require ethical approval and should be discussed with the authors by contacting the corresponding author. Regarding propriety rights of study material and results, the hospitals are the owner of the raw data of their hospital. Sciensano and the hospitals are the owner of the analysed data, aggregated tables and inferential results.
