# Supplementary material for: Evaluation of the added value of viral genomic information for predicting severity of influenza infection
Source: BMC Infect Dis. 2021 Aug 10;21:785. doi: 10.1186/s12879-021-06510-z (PMC8353062; doi:10.1186/s12879-021-06510-z)
Supplement: Supplementary file 1 — Additional file 1. Appendix A: Internal validation. Appendix B: Exploration of other data analysis scenarios. Appendix C: Comparison baseline characteristics to assess selection bias. Appendix D: List of 25 mutations with an unadjusted p-value below 0.05. Appendix E: Manhattan plot. Appendix F: Principle Component Analysis (PCA). Appendix G: Apparent Area Under the ROC curve. Appendix H: Boxplots of the fitted values for the host and the combined model. Appendix I: Genomic model. [file 12879_2021_6510_MOESM1_ESM.docx]

**Evaluation of the added value of viral genomic information for predicting severity of influenza infection**

**Supplementary Appendix**

Nina Van Goethem, Annie Robert, Nathalie Bossuyt, Laura Van Poelvoorde, Sophie Quoilin, Sigrid C. J. De Keersmaecker, Brecht Devleesschauwer, Isabelle Thomas, Kevin Vanneste, Nancy H. C. Roosens^*^, Herman Van Oyen^*^

*Shared last author

Corresponding author: [nina.vangoethem@sciensano.be](mailto:nina.vangoethem@sciensano.be)

**List of Supplementary Appendices**

**A: Internal validation**

**B: Exploration of other data analysis scenarios**

**C: Comparison baseline characteristics to assess selection bias**

**D: List of 25 mutations with an unadjusted p-value below 0.05**

**E: Manhattan plot**

**F: Principle Component Analysis (PCA)**

**G: Apparent Area Under the ROC curve**

**H: Boxplots of the fitted values for the host and the combined model**

**I: Genomic model**

**Supplementary Appendix A: Internal validation**

The apparent Area Under the Receiver Operating Characteristic curve (AUC) is based on the same dataset that was used for model building and will be optimistically biased [1–6] and therefore be prone to overfitting so that prediction models should always undergo validation. As the availability of additional data is often limited, internal validation is widely considered as an approximation of external validation [1, 2, 4, 5, 7]. A first approach is to split the available data into a ‘training’ and a ‘test’ set for respectively generating and testing models on independent datasets. Such ‘split-sample’ validation requires however a still relatively large sample size [2]. If the test dataset size is small, the estimate of the out-sample error may show a large variance and hence may not be reliable. Additionally, a lower AUC on the test compared to the training dataset cannot only be caused by overfitting, but also be due to other random differences between both datasets, especially when sample sizes are small. Moreover, model evaluation for a relatively infrequent outcome (such as severity) based on split-sample validation has been reported to underestimate the true predictive performance [1, 2]. A second approach, i.e. the one that we adopted in the current study, is internal validation through bootstrapping (optimism-adjusted) [2, 8] to obtain an internally validated AUC, sensitivity and specificity. Optimism-adjusted accuracy measures take into account the estimated deterioration that the model will experience when applied to new subjects [9], and resampling approaches provide unbiased model assessment without reducing the data set size for training the model. A disadvantage to internal validation is that it does not reflect many of the sources of variability present in applying a prediction model in broad clinical practice outside of the study conditions [6], for which external validation would be more appropriate [2]. It should also be noted that the model building process itself (i.e. selection of predictors) was not included in the model validation as not all modeling decisions could be systematically replayed, but a penalized regression methodology already incorporating cross-validation and bootstrapping was applied to the data.

**Supplementary Appendix B: Exploration of other data analysis scenarios**

Instead of performing a penalized elastic net regression on the full dataset, a sub-selection of covariates was made based on univariate analyses (i.e. prioritization). As such, factors found to be associated significantly with severity in univariate analysis were included in the multivariable analysis (with a significance level α = 0.10). Stepwise regression with both forward and backward selection was used to choose covariates to be conserved in the model.


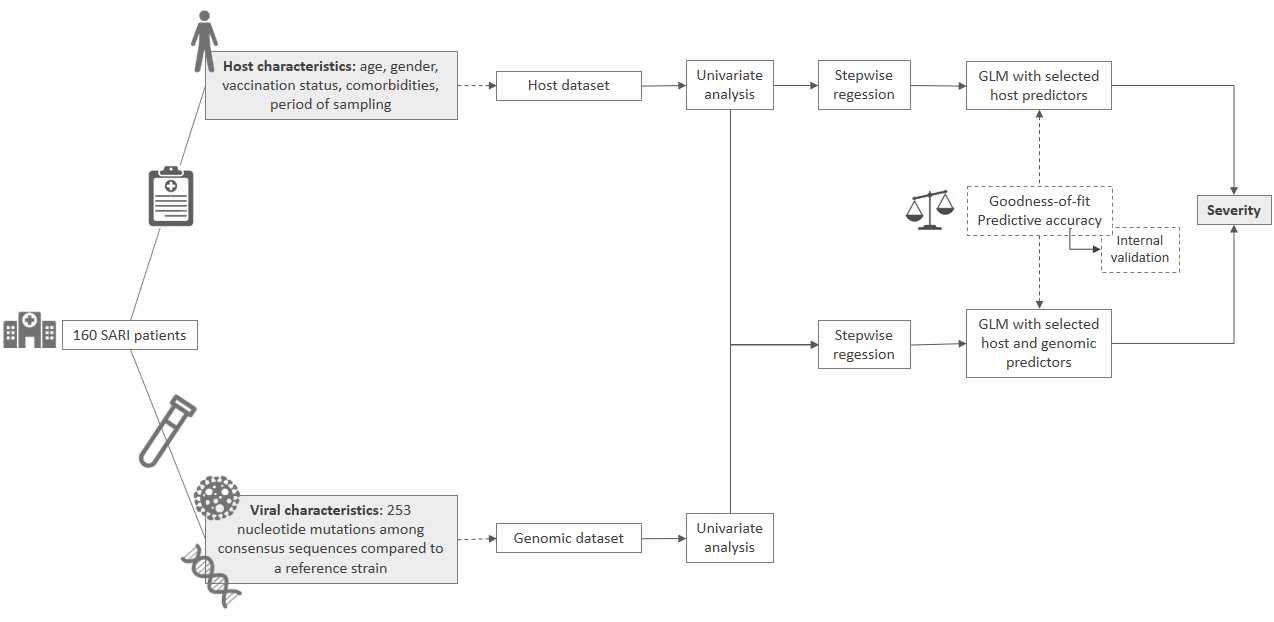


**Figure 1.** Overview of the predictive model building process to compare a model including variables obtained from a combined dataset (i.e. host characteristics and viral characteristics) and a model including variables from the host characteristics dataset. SARI = Severe Acute Respiratory Infection. GLM = Generalized Linear Model.

The following host covariates were considered for multivariable analysis: chronic cardiovascular condition (p=0.047, Fisher’s exact test), chronic respiratory condition (p=0.018, Fisher’s exact test), immunocompromised status (p=0.015, Fisher’s exact test), and renal insufficiency (p=0.023, Fisher’s exact test). A stepwise regression based on these host characteristics resulted in the following model, as presented in Table 1, with an AIC of 168.63.

**Table 1.** Host factors predicting severity of influenza.

| **Variable** | **Odds ratio** | **95% CI** |
| --- | --- | --- |
| **Chronic respiratory condition** | 2.16 | [0.93, 4.95] |
| **Renal insufficiency** | 1.94 | [0.78, 4.64] |
| **Immunocompromised disorder** | 3.00 | [1.09, 8.19] |
| **Chronic cardiovascular condition** | 2.05 | [0.91, 4.61] |

Following a Fisher’s exact test to test the association between the mutations and severity, 58 mutations had a (unadjusted) p-value below 0.10. Eventually, stepwise regression based on the 4 host characteristics and 58 mutations selected to following covariates for the combined model, as presented in Table 2, with an AIC of 157.64 (covariates with inflated standard errors were removed from the final model).

**Table 2.** Host factors and mutations (from the combined dataset) predicting severity of influenza.

| **Variable** | **Odds ratio** | **95% CI** |
| --- | --- | --- |
| **Chronic respiratory condition** | 3.31 | [1.32, 8.54] |
| **Renal insufficiency** | 2.30 | [0.86, 6.12] |
| **Immunocompromised disorder** | 3.59 | [1.19, 11.07] |
| **PA T135C** | 0.18 | [0.01, 1.02] |
| **PA G1089A** | 2.39 | [0.62, 9.32] |
| **PA A1475G** | 3.85 | [0.76, 20.04] |
| **NA C1005T** | 3.17 | [1.19, 8.86] |
| **NS A323C** | 5.94 | [1.50, 23.97] |

Subsequently, the combined model was compared to a model only including the host characteristics. The likelihood-ratio test requires that the models are nested. Both models did not select exactly the same set of host characteristics: the combined model did not select chronic cardiovascular condition. Adding the 5 selected mutations (PA T135C, PA G1089A, PA A1475G, NA C1005T, and NS A323C) to 3 host characteristics (chronic respiratory condition, renal insufficiency, and immunocompromised condition) significantly improved the model fit ($X^{2}$= 21.97, df=5, p=0.0005). Similarly, adding the 5 selected mutations to 4 host characteristics (chronic respiratory condition, renal insufficiency, immunocompromised condition, and chronic cardiovascular condition) significantly improved the model fit ($X^{2}$= 21.58, df=5, p=0.0006).

The apparent AUC of the host model (Table 1) was equal to 0.700 [0.602,0.799], whereas the combined model (Table 2) has an apparent AUC of 0.814 [0.738,0.891]. Statistical comparison of the apparent AUC of the models resulted in a p-value of 0.01. The optimism-adjusted measures of predictive accuracy are presented in Table 3.

**Table 3.** Bootstrap (using 200 resamples) optimism-corrected measures of accuracy for both models.

|  | **Combined model** | | | **Host model** | | |
| --- | --- | --- | --- | --- | --- | --- |
|  | **Corrected** | **Optimism** | **Apparent** | **Corrected** | **Optimism** | **Apparent** |
| **AUC** | 0.770 | 0.044 | 0.814 | 0.670 | 0.030 | 0.700 |

It should be noted that a stepwise regression assumes that there exists no (multi)collinearity between the predictors. As a lot of mutations are expected to be highly correlated, the elastic net approach is considered more appropriate.

**Supplementary Appendix C: Comparison baseline characteristics to assess selection bias**

**Table 1**. Comparison baseline characteristics between the full set of influenza positive severe SARI* patients, without co-infection, present in the surveillance dataset (n=50) and the full set of influenza positive non-severe SARI* patients, without co-infection, present in the surveillance dataset (n=345), Belgium, Influenza season 2016-2017.

|  | **All SARI influenza positive H3N2 16-17 severe patients (n=50)** | | **All SARI influenza positive H3N2 16-17 non-severe patients (n=345)** |  | **p value**^§^ |
| --- | --- | --- | --- | --- | --- |
|  | *Median + IQR^⁋^* | *Total* | *Median + IQR^⁋^* | *Total* |  |
| **Age** | 77 (67-82) | 49 | 76 (56-84) | 341 | 0.47 |
| **Period of sampling in weeks** | 5 (4-6) | 50 | 5 (4-6) | 345 | 0.88 |
|  | *Proportion* | *Total* | *Proportion* | *Total* |  |
| **Males** | 0.50 | 48 | 0.43 | 329 | 0.36 |
| **Vaccinated** | 0.52 | 27 | 0.38 | 217 | 0.21 |
| **Chronic cardiovascular condition** | 0.44 | 50 | 0.28 | 345 | 0.03 |
| **Chronic respiratory condition** | 0.44 | 50 | 0.21 | 345 | 0.001 |
| **Renal insufficiency** | 0.30 | 50 | 0.15 | 345 | 0.01 |
| **Hepatic insufficiency** | 0.02 | 50 | 0.03 | 345 | > 0.99 |
| **Immunocompromised condition** | 0.30 | 50 | 0.14 | 345 | 0.01 |
| **Pregnant** | 0.00 | 50 | 0.02 | 345 | / |
| **Asthma** | 0.02 | 50 | 0.06 | 345 | 0.49 |
| **Neuromuscular condition** | 0.08 | 50 | 0.10 | 345 | 0.80 |
| **Diabetes** | 0.20 | 50 | 0.16 | 345 | 0.54 |
| **Obesity** | 0.08 | 50 | 0.10 | 345 | 0.80 |

^*^: SARI = Severe acute respiratory infection.

*^⁋^*: IQR = inter-quartile range.

^§^: Wilcoxon rank sum test applied for medians and Fisher’s exact test applied for proportions.

**Supplementary Appendix D: List of 25 mutations with an unadjusted p-value below 0.05**

**Table 1**. Association between mutations and severity, list of 25 mutations with an unadjusted p-value below 0.05, SARI surveillance season 2016-2017, Belgium.

| **Nucleotide mutation** | **P value**^§^ |
| --- | --- |
| NP C1236T | 0.01130008670 |
| NS T471C | 0.01130008670 |
| NA T446C | 0.01447864314 |
| NP G506A | 0.01653759380 |
| PB1 A2043G | 0.01855120331 |
| NA T1011C | 0.01855120331 |
| MP A871G | 0.01855120331 |
| NS A336G | 0.01855120331 |
| HA A1197G | 0.01892631225 |
| NP T1176C | 0.02789944361 |
| PA T135C | 0.02804973681 |
| PB2 A1389G | 0.02925317638 |
| PA C96T | 0.02925317638 |
| NP G1153A | 0.02979878616 |
| NS A586G | 0.02979878616 |
| PB2 G896A | 0.03011996033 |
| NP A527G | 0.03053318804 |
| NA C418A | 0.03299797592 |
| PA C1776T | 0.03681211111 |
| PB1 A633G | 0.04248867656 |
| NA T85C | 0.04248867656 |
| PA C978T | 0.04278977904 |
| PB1 C915T | 0.04621736951 |
| PA G1056A | 0.04621736951 |
| NA A1095G | 0.04621736951 |

^§^: Unadjusted p-values following a Fisher’s exact test.

**Supplementary Appendix E: Manhattan plot**


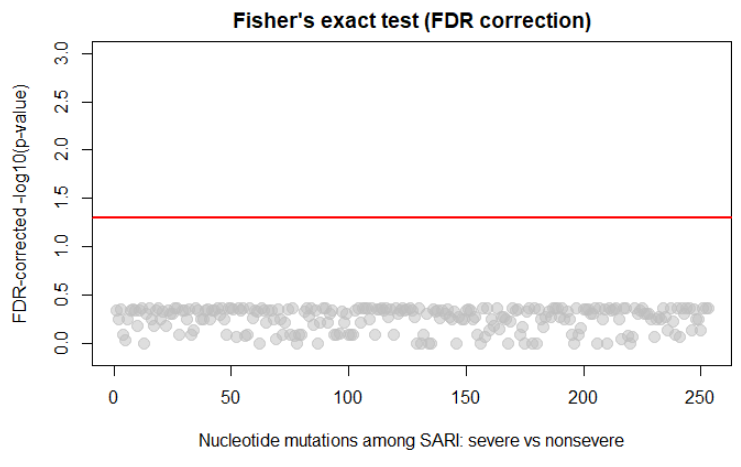


**Figure 1.** Manhattan plot of the 253 consensus mutations. The Y-axis presents the negative logarithm of the FDR-corrected q-value of the association between mutations and severity. The red horizontal line indicates the 5% FDR-threshold. No significant associations could be identified between mutations and severity. FDR = false discovery rate. SARI = severe acute respiratory infection.

**Supplementary Appendix F: Principle Component Analysis (PCA)**


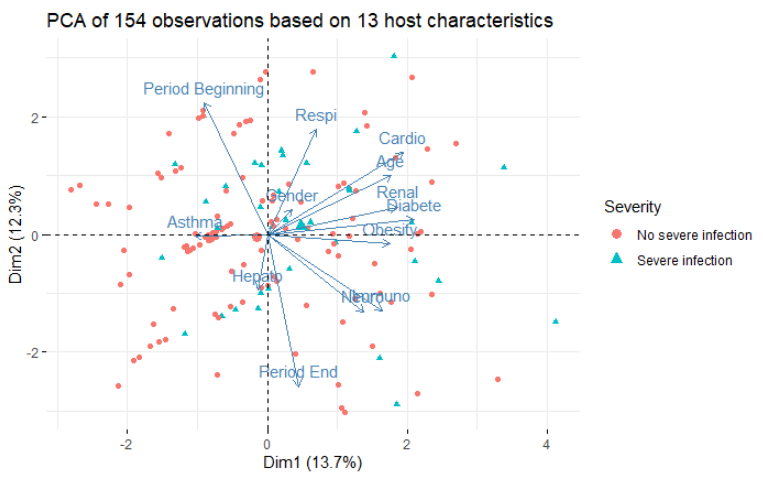


**Figure 1. Biplot of 154 observations based on 13 host characteristics.** A principal component analysis (PCA) was constructed based on the following variables: period of sampling during the influenza season (beginning, middle, end), age, gender, and the co-morbidities (chronic respiratory condition, chronic cardiovascular condition, renal insufficiency, hepatic insufficiency, immunocompromised condition, pregnancy, asthma, neuromuscular condition, diabetes, and obesity). The two pregnant women were excluded to perform the PCA, as pregnancy is correlated with gender and these two patients appeared to be outliers. Further, 4 patients for which the gender was unknown were excluded as well, leaving a total of 154 patients that were included in the PCA. Only 26% of the variation in the data is explained by the first two principal components (dimensions). Dim1: first dimension. Dim2: second dimension.


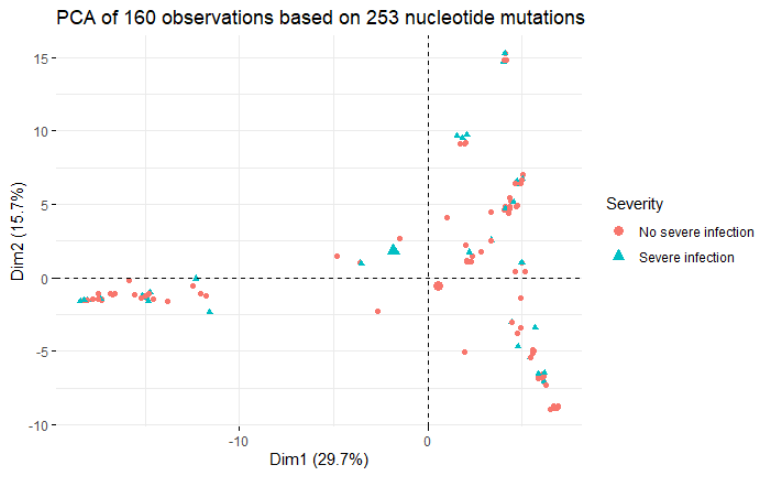


**Figure 2. Graph of 160 observations based on 253 mutations.** A Principal Component Analysis (PCA) was performed based on the 253 nucleotide mutations to examine the sources of variation in the genomic data. A graph of the observations is constructed according to the first two dimensions. The observations are colored according to severity to visualize potential phenotypic clusters along the significant axes of PCA and to assess the extent of population stratification. 45% of variation in the data is captured by the first two principal components (dimensions). Dim1: first dimension. Dim2: second dimension.

**Supplementary Appendix G: Apparent Area Under the ROC curve**


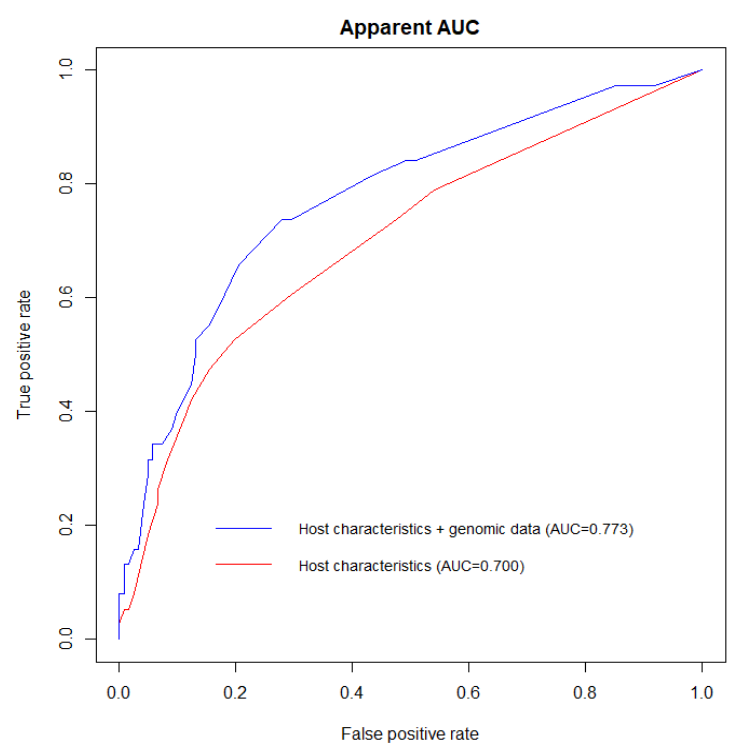


**Figure 1.** Apparent (or in-sample) area under the Receiver Operating Characteristic (ROC) curve (AUC) of the predictive models.

**Supplementary Appendix H: Boxplots of the fitted values for the host and the combined model**


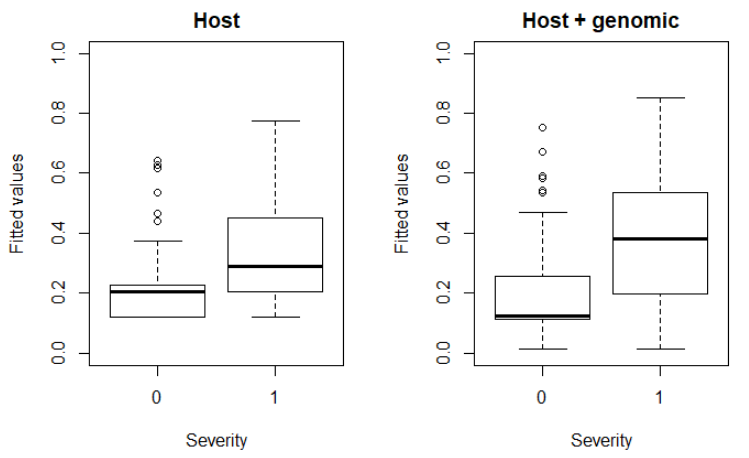


**Figure 1. Boxplots of the fitted values for both models.** 1 = severe, 0 = not severe.

**Supplementary Appendix I: Genomic model**

The same elastic net approach, as was described in the methods for the host dataset and the combined dataset, was applied to the genomic dataset (i.e. including 253 mutations). The parameter estimates of the elastic net model with host characteristics as input were alpha = 0.4 and lambda = 0.018. The following mutations were selected: PA A1475G, HA G473A, NP G506A, and NS A323C.


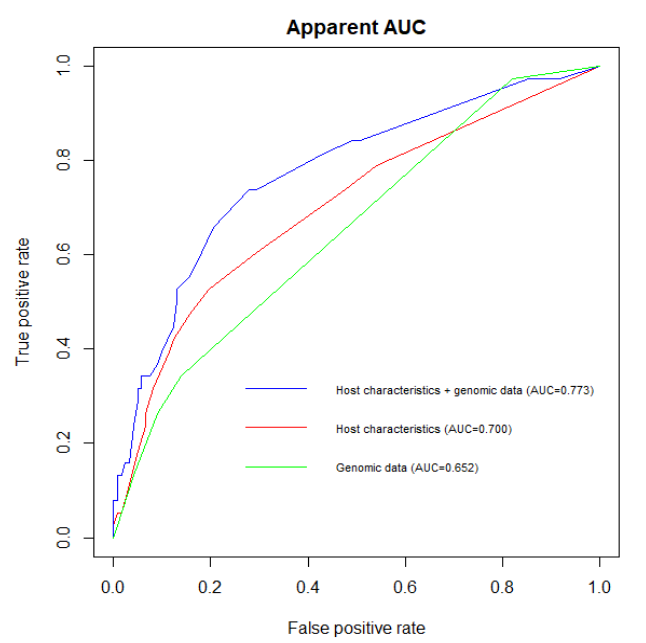


**Figure 1.** Apparent (or in-sample) area under the Receiver Operating Characteristic (ROC) curve (AUC) of the predictive models.

**References for Supplementary Appendices**

1. Mondol MH, Rahman MS. A comparison of internal validation methods for validating predictive models for binary data with rare events. Journal of Statistical Research. 2017;51:131–44.

2. Steyerberg EW, Harrell FE, Borsboom GJ, Eijkemans MJ, Vergouwe Y, Habbema JD. Internal validation of predictive models: efficiency of some procedures for logistic regression analysis. J Clin Epidemiol. 2001;54:774–81.

3. Sainani KL. Explanatory Versus Predictive Modeling. PM&R. 2014;6:841–4.

4. Giancristofaro RA, Salmaso L. Model performance analysis and model validation in logistic regression. Stat. 2003;63:375–96.

5. Smith GCS, Seaman SR, Wood AM, Royston P, White IR. Correcting for Optimistic Prediction in Small Data Sets. Am J Epidemiol. 2014;180:318–24.

6. Simon RM, Subramanian J, Li M-C, Menezes S. Using cross-validation to evaluate predictive accuracy of survival risk classifiers based on high-dimensional data. Brief Bioinform. 2011;12:203–14.

7. Justice AC, Covinsky KE, Berlin JA. Assessing the generalizability of prognostic information. Ann Intern Med. 1999;130:515–24.

8. Harrell FE, Lee KL, Mark DB. Multivariable prognostic models: issues in developing models, evaluating assumptions and adequacy, and measuring and reducing errors. Stat Med. 1996;15:361–87.

9. Faraklas I, Stoddard GJ, Neumayer LA, Cochran A. Development and validation of a necrotizing soft-tissue infection mortality risk calculator using NSQIP. J Am Coll Surg. 2013;217:153-160.e3; discussion 160-161.
